# Supplementary material for: Simultaneous Analysis and Dietary Exposure Risk Assessment of Fomesafen, Clomazone, Clethodim and Its Two Metabolites in Soybean Ecosystem
Source: Int J Environ Res Public Health. 2020 Mar 17;17(6):1951. doi: 10.3390/ijerph17061951 (PMC7143629; doi:10.3390/ijerph17061951)
Supplement: Supplementary file 1 [file ijerph-17-01951-s001.pdf]

International Journal of
Environmental Research and Public Health

MDPI

Supplementary Material

Simultaneous Analysis and Dietary Exposure risk Assessment of Fomesafen, Clomazone, Clethodim and its Two Metabolites in Soybean Ecosystem

Kyongjin Pang and Jiye Hu*

School of Chemistry and Biological Engineering, University of Science Technology Beijing, Beijing, 100083, PR China

Address: Lab of Pesticide Residues and Environmental Toxicology, School of Chemistry and Biological Engineering, University of Science and Technology Beijing, 30 Xueyuan Road, Haidian District, Beijing 100083, China

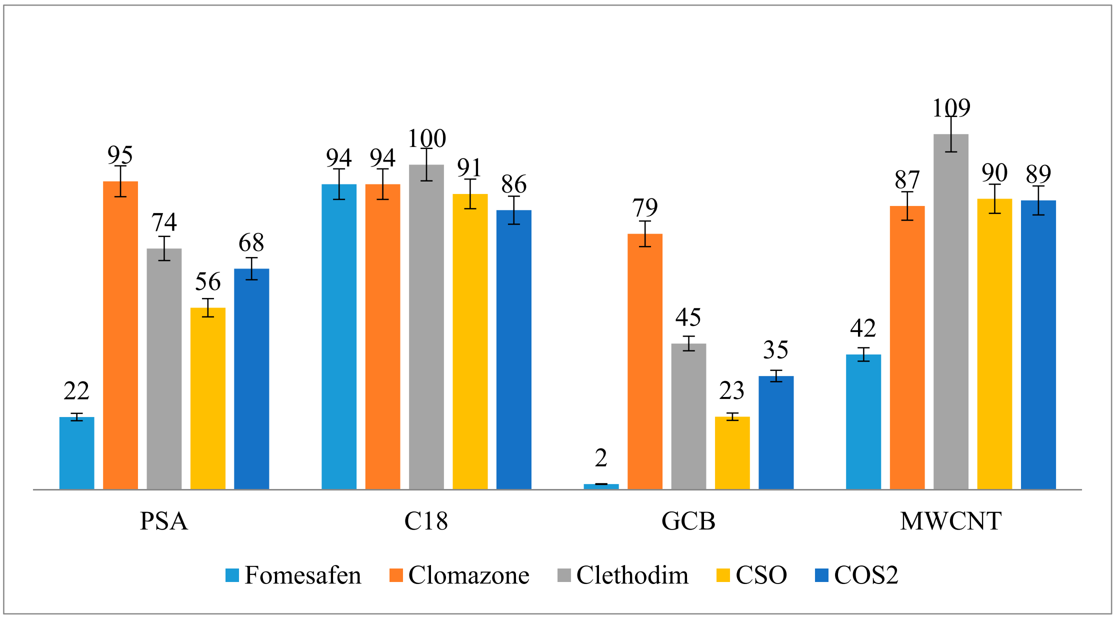

Figure 1. Recoveries (%) of fomesafen, clomazone, clethodim, CSO and CSO_2 in soybean matrix using varous d-SPE.

* Corresponding author. Tel: +86 82376002 Fax: +86 1082376002

E-mail address: jyhu@ustb.edu.cn (Jiye Hu) , banggyongjin@163.com (Kyongjin Pang)

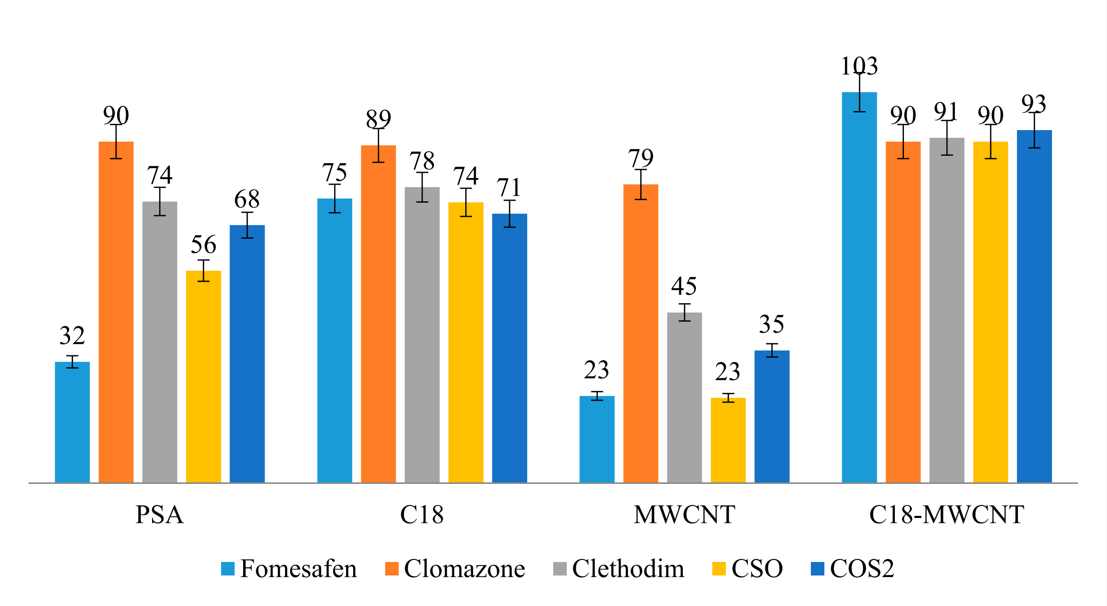

Figure 2. Recoveries (%) of fomesafen, clomazone, clethodim, CSO and CSO_2 in green soybean matrix using various d-SPE.

Table 1. Properties of soil and climate conditions in different field trial sites.

**Sites**	**pH value of soils**	**Organic matter content of soils (%)**	**Average temperature (°C)**	**Rainfalls（mm）**	**East longitude, Northern latitude**
Heilongjiang	6.90	3.2	22.4	152	128°45' E, 45°05' N
Liaoning	7.10	3.4	26.3	500	123°25' E, 41°48′ N
Inner molgolia	7.20	2.7	23.7	550	113°07' E, 40°59' N
Shanxi	7.20	1.9	24.0	300	110°15' E, 35°49' N
Anhui	7.4	1.2	28.1	1270	116°93' E, 34°19' N
Guangxi	7.1	1.4	25.2	2208	108°21' E, 22°49' N

Table 2. LODs linear relationships, and repeatabilities (n = 6) for determination of three herbicides in different matrices.

compound	matrix	Matrix matched calibration	correlation coefficient (R^2)	Matrix effect (%)
Fomesafen	Soybean	y = 8057.9x + 117.33	0.9904	13
	Green Soybean	y = 3154.6x - 10.634	0.9996	-1.2
	Soybean Straw	y = 4306.8x + 1.3098	0.9997	0.9
Clomazon e	Soybean	y = 74881x - 43.316	0.9996	-6.2
	Green Soybean	y = 45304x - 33.917	0.9998	-5.8
	Soybean Straw	y = 64759x - 222.85	0.9995	-11
Clethodim	Soybean	y = 42386x + 1410.3	0.9932	10
	Green Soybean	y = 20250x + 248.78	0.9998	9
	Soybean Straw	y = 37915x - 30.726	0.9994	-3.1
CSO	Soybean	y = 216191x + 2060.3	0.9966	10
	Green Soybean	y = 54838x + 282.88	0.9999	13
	Soybean Straw	y = 113576x + 230.81	1	11
CSO_2	Soybean	y = 50382x + 131.49	0.9996	8
	Green Soybean	y = 22006x + 100.57	0.9999	14
	Soybean Straw	y = 39572x + 123.32	0.9999	11

Table 3. Terminal residues of fomesafen in soybean.

Site of field trial	Sprayed dosage （g a.i. /ha）	Number of Application	Interval of application （d）	Terminal residues（mg/kg）		
				1	2	Average
Heilongjiang	721.5	1	118	<0.01	<0.01	<0.01
Inner Molgolia	721.5	1	105	<0.01	<0.01	<0.01
Shanxi	721.5	1	98	<0.01	<0.01	<0.01
Liaoning	721.5	1	120	<0.01	<0.01	<0.01
Guangxi	721.5	1	109	<0.01	<0.01	<0.01
Anhui	721.5	1	50	<0.01	<0.01	<0.01

Table 4. Terminal residues of Clomazone in soybean.

Site of field trial	Sprayed dosage (g a.i. /ha)	Number of Application	Interval of application (d)	Terminal residues (mg/kg)		
				1	2	Average
Heilongjiang	721.5	1	118	<0.01	<0.01	<0.01
Inner Molgolia	721.5	1	105	<0.01	<0.01	<0.01
Shanxi	721.5	1	98	<0.01	<0.01	<0.01
Liaoning	721.5	1	120	<0.01	<0.01	<0.01
Guangxi	721.5	1	109	<0.01	<0.01	<0.01
Anhui	721.5	1	50	<0.01	<0.01	<0.01

Table 5. Terminal residues of Clethodim in soybean.

Site of field trial	Sprayed dosage (g a.i. /ha)	Number of Application	Interval of application (d)	Terminal residues (mg/kg)		
				1	2	Average
Heilongjiang	721.5	1	118	<0.01	<0.01	<0.01
Inner Molgolia	721.5	1	105	<0.01	<0.01	<0.01
Shanxi	721.5	1	98	<0.01	<0.01	<0.01
Liaoning	721.5	1	120	<0.01	<0.01	<0.01
Guangxi	721.5	1	109	<0.01	<0.01	<0.01
Anhui	721.5	1	50	<0.01	<0.01	<0.01

Table 6. Terminal residues of CSO in soybean.

Site of field trial	Sprayed dosage (g a.i. /ha)	Number of Application	Interval of application (d)	Terminal residues (mg/kg)		
				1	2	Average
Heilongjiang	721.5	1	118	<0.01	<0.01	<0.01
Inner Molgolia	721.5	1	105	<0.01	<0.01	<0.01
Shanxi	721.5	1	98	<0.01	<0.01	<0.01
Liaoning	721.5	1	120	<0.01	<0.01	<0.01
Guangxi	721.5	1	109	<0.01	<0.01	<0.01
Anhui	721.5	1	50	<0.01	<0.01	<0.01

Table 7. Terminal residues of CSO_2 in soybean.

Site of field trial	Sprayed dosage (g a.i. /ha)	Number of Application	Interval of application (d)	Terminal residues (mg/kg)		
				1	2	Average
Heilongjiang	721.5	1	118	<0.01	<0.01	<0.01
Inner Molgolia	721.5	1	105	<0.01	<0.01	<0.01
Shanxi	721.5	1	98	<0.01	<0.01	<0.01
Liaoning	721.5	1	120	<0.01	<0.01	<0.01
Guangxi	721.5	1	109	<0.01	<0.01	<0.01
Anhui	721.5	1	50	<0.01	<0.01	<0.01

Table 8. Terminal residues of fomesafen in green soybean.

Site of field trial	Sprayed dosage（g a.i. /ha）	Number of application	Interval of application（d）	Terminal residues（mg/kg）		
				1	2	Average
Heilongjiang	721.5	1	89	<0.01	<0.01	<0.01
Inner Molgolia	721.5	1	76	<0.01	<0.01	<0.01
Shanxi	721.5	1	73	<0.01	<0.01	<0.01
Liaoning	721.5	1	87	<0.01	<0.01	<0.01
Guangxi	721.5	1	82	<0.01	<0.01	<0.01
Anhui	721.5	1	22	<0.01	<0.01	<0.01

Table 9. Terminal residues of Clomazone in green soybean.

Site of field trial	Sprayed dosage（g a.i. /ha）	Number of application	Interval of application（d）	Terminal residues（mg/kg）		
				1	2	Average
Heilongjiang	721.5	1	89	<0.01	<0.01	<0.01
Inner Molgolia	721.5	1	76	<0.01	<0.01	<0.01
Shanxi	721.5	1	73	<0.01	<0.01	<0.01
Liaoning	721.5	1	87	<0.01	<0.01	<0.01
Guangxi	721.5	1	82	<0.01	<0.01	<0.01
Anhui	721.5	1	22	<0.01	<0.01	<0.01

Table 10. Terminal residues of Clethodim in green soybean.

Site of field trial	Sprayed dosage（g a.i. /ha）	Number of application	Interval of application（d）	Terminal residues（mg/kg）		
				1	2	Average
Heilongjiang	721.5	1	89	<0.01	<0.01	<0.01
Inner Molgolia	721.5	1	76	<0.01	<0.01	<0.01
Shanxi	721.5	1	73	<0.01	<0.01	<0.01
Liaoning	721.5	1	87	<0.01	<0.01	<0.01
Guangxi	721.5	1	82	<0.01	<0.01	<0.01
Anhui	721.5	1	22	<0.01	<0.01	<0.01

Table 11. Terminal residues of CSO in green soybean.

Site of field trial	Sprayed dosage（g a.i. /ha）	Number of application	Interval of application（d）	Terminal residues（mg/kg）		
				1	2	Average
Heilongjiang	721.5	1	89	<0.01	<0.01	<0.01
Inner Molgolia	721.5	1	76	<0.01	<0.01	<0.01
Shanxi	721.5	1	73	<0.01	<0.01	<0.01
Liaoning	721.5	1	87	<0.01	<0.01	<0.01
Guangxi	721.5	1	82	<0.01	<0.01	<0.01
Anhui	721.5	1	22	<0.01	<0.01	<0.01

Table 12. Terminal residues of CSO_2 in green soybean.

Site of field trial	Sprayed dosage（g a.i. /ha）	Number of application	Interval of application（d）	Terminal residues（mg/kg）		
				1	2	Average
Heilongjiang	721.5	1	89	<0.01	<0.01	<0.01
Inner Molgolia	721.5	1	76	<0.01	<0.01	<0.01
Shanxi	721.5	1	73	<0.01	<0.01	<0.01
Liaoning	721.5	1	87	<0.01	<0.01	<0.01
Guangxi	721.5	1	82	<0.01	<0.01	<0.01
Anhui	721.5	1	22	0.015	0.014	0.015

Table 13. Terminal residues of fomesafen in soybean straw.

Site of field trial	Sprayed dosage（g a.i. /ha）	Number of application	Interval of application（d）	Terminal residues（mg/kg）		
				1	2	Average
Heilongjiang	721.5	1	118	<0.02	<0.02	<0.02
Inner Molgolia	721.5	1	105	<0.02	<0.02	<0.02
Shanxi	721.5	1	98	<0.02	<0.02	<0.02
Liaoning	721.5	1	120	<0.02	<0.02	<0.02
Guangxi	721.5	1	109	<0.02	<0.02	<0.02
Anhui	721.5	1	50	<0.02	<0.02	<0.02

Table 14. Terminal residues of Clomazone in soybean straw.

Site of field trial	Sprayed dosage（g a.i. /ha）	Number of application	Interval of application（d）	Terminal residues（mg/kg）		
				1	2	Average
Heilongjiang	721.5	1	118	<0.02	<0.02	<0.02
Inner Molgolia	721.5	1	105	<0.02	<0.02	<0.02
Shanxi	721.5	1	98	<0.02	<0.02	<0.02
Liaoning	721.5	1	120	<0.02	<0.02	<0.02
Guangxi	721.5	1	109	<0.02	<0.02	<0.02
Anhui	721.5	1	50	<0.02	<0.02	<0.02

Table 15. Terminal residues of Clethodim in soybean straw.

Site of field trial	Sprayed dosage（g a.i. /ha）	Number of application	Interval of application（d）	Terminal residues（mg/kg）		
				1	2	Average
Heilongjiang	721.5	1	118	<0.02	<0.02	<0.02
Inner Molgolia	721.5	1	105	<0.02	<0.02	<0.02
Shanxi	721.5	1	98	<0.02	<0.02	<0.02
Liaoning	721.5	1	120	<0.02	<0.02	<0.02
Guangxi	721.5	1	109	<0.02	<0.02	<0.02
Anhui	721.5	1	50	<0.02	<0.02	<0.02

Table 16. Terminal residues of CSO in soybean straw.

**Site of field trial**	**Sprayed dosage (g a.i. /ha)**	**Number of application**	Interval of application (d)	Terminal residues (mg/kg)		
				1	2	Average
Heilongjiang	721.5	1	118	<0.02	<0.02	<0.02
Inner Molgolia	721.5	1	105	<0.02	<0.02	<0.02
Shanxi	721.5	1	98	<0.02	<0.02	<0.02
Liaoning	721.5	1	120	<0.02	<0.02	<0.02
Guangxi	721.5	1	109	<0.02	<0.02	<0.02
Anhui	721.5	1	50	<0.02	<0.02	<0.02

Table 17. Terminal residues of CSO_2 in soybean.

**Site of field trial**	**Sprayed dosage (g a.i. /ha)**	**Number of application**	Interval of application (d)	Terminal residues (mg/kg)		
				1	2	Average
Heilongjiang	721.5	1	118	<0.02	<0.02	<0.02
Inner Molgolia	721.5	1	105	<0.02	<0.02	<0.02
Shanxi	721.5	1	98	<0.02	<0.02	<0.02
Liaoning	721.5	1	120	<0.02	<0.02	<0.02
Guangxi	721.5	1	109	0.030	0.028	0.029
Anhui	721.5	1	50	<0.02	<0.02	<0.02

Table 18. Recoveries, RSDs and LOQs of five substances (fomesafen, clomazone, clethodim, CSO and CSO2) in several matrics by previous study.

**Compounds**	**Matrix**	**Recovery (%)**	**RSDs (%)**	**LOQs (mg/kg)**	**LODs (μg/kg)**	**Equipement**	**pretreatment**
Fomesafen	Tomato [1]	99-102	4.2	0.001	0.0015	LC-MS/MS	QuEChERS
	Soil [2]	83-102	≤4.02	0.05		HPLC-DAD	Liquid-liquid
	Plant [2]	85-99	3.51	0.05		HPLC-DAD	Liquid-liquid
	Earthworm [2]	84-91	3.29	0.05		HPLC-DAD	Liquid-liquid
	Juice [6]	96-98	3.7	0.297 mg/L	0.089 mg/L	Cyclic Voltammetry	Liquid-liquid
	Leek [10]	76.3-88.4	18	0.01	10	LC-MS/MS	QuEChERS
Clomazone	River water [11]	87.3-104	≤4.07	0.1 μg/L		HPLC-DAD	Liquid-liquid
	Soil [9]	87-92	8.78	0.01	0.25	LC-MS/MS	QuEChERS
	Soybean [3]	96-97	4.9	0.01		LC-MS/MS	Liquid-liquid
	Soybean [4]	94-106	6	0.01		LC-MS/MS	QuEChERS
	Green soybean [4]	89-109	11	0.01		LC-MS/MS	QuEChERS
	Soybean straw [4]	91-106	3	0.01		LC-MS/MS	QuEChERS
	Leek [10]	95-95	8.1	0.01	1	LC-MS/MS	QuEChERS
Clethodim	radish, tomato, onion, sweet	91-118	≤10	0.05	10	LC-MS/MS	Liquid-liquid

	potato, kidney bean, carrot, cabbage, lettuce [7]						
	Rape plant [5]	85-90.1	≤6.9	0.01	4	LC-MS/MS	QuEChERS
	Rape seed [5]	84.6-96.7	≤5.4	0.05	10	LC-MS/MS	QuEChERS
	green tobacco leaf [8]	78.3-88.1	7.0	0.08	0.024	LC-MS/MS	QuEChERS
	cured tobacco leaf [8]	93.9-94.6	6.8	0.2	0.06	LC-MS/MS	QuEChERS
CSO	Rape plant [5]	80.3-84.9	≤5.4	0.01	3	LC-MS/MS	QuEChERS
	Rape seed [5]	78.7-101.6	≤7.6	0.05	10	LC-MS/MS	QuEChERS
	green tobacco leaf [8]	74.8–104.4	≤3.4	0.08	0.024	LC-MS/MS	QuEChERS
	cured tobacco leaf [8]	87.6-105.5	≤5.7	0.2	0.06	LC-MS/MS	QuEChERS
CSO_2	Rape plant [5]	83.6-84.8	≤3.9	0.01	3	LC-MS/MS	QuEChERS
	Rape seed [5]	91.6-102.8	≤7.7	0.05	10	LC-MS/MS	QuEChERS
	green tobacco leaf [8]	89.2-96.7	≤6.2	0.08	0.024	LC-MS/MS	QuEChERS
	cured tobacco leaf [8]	89.3-102.0	≤5.0	0.2	0.06	LC-MS/MS	QuEChERS

References

[1] Li, Z.; Di Gioia, F.; Hwang, J.; Hong, J.; Ozores-Hampton, M.; Zhao, X.; Pisani, C.; Rosskopf, E.; Wilson, P.C. Dissipation of fomesafen in fumigated, anaerobic soil disinfestation-treated, and organic-amended soil in Florida tomato production systems, *Pest. Manag. Sci.* **2019**. https://doi.org/10.1002/ps.5558.

[2] Khorram, M.S.; Zheng, Y.; Lin, D.; Zhang, Q.; Fang, H.; Yu, Y. Dissipation of fomesafen in biochar-amended soil and its availability to corn (Zea mays L.) and earthworm (Eisenia fetida), *J Soil. Sedimet.* **2016**, 16, 2439-2448.

[3] Hu, J.Y.; Cao, D.; Deng, Z.B. Determination of clomazone residues in soybean and soil by high performance liquid chromatography with DAD detection, *B. Environ. Contam. Tox.* **2011**, 86, 444-448.

[4] Hussan, H.N.M.; He, H.R.; Du, P.Q.; Wu, X.H.; Liu, X.G.; Xu, J.; Dong, F.S.; Zheng, Y.Q. Determination of clomazone and acetochlor residues in soybean (Glycine max (L.) Merr.), *Int. J. Environ. An. Ch.* **2020**, DOI: 10.1080/03067319.2019.1694668.

[5] You, X.; Liang, L.; Liu, F. Dissipation and residues of clethodim and its oxidation metabolites in a rape-field ecosystem using QuEChERS and liquid chromatography/tandem mass spectrometry, *Food. Chem.* **2014**, 43, 170-174.

[6] Demir, E.; Inam, R. Square Wave Voltammetric Determination of Fomesafen Herbicide Using Modified Nanostructure Carbon Paste Electrode as a Sensor and Applicaion to Food Samples, *Food. Anal. Method.* **2017**, 10, 74-82.

[7] Ishimitsu, S.; Kaihara, A.; Yoshii, K.; Tsumura, Y.; Nakamura, Y.; Tonogai, Y. Determination of clethodim and its oxidation metabolites in crops by liquid chromatography with confirmation by LC/MS, *J. Aoac. Int.* **2001**, 84, 1172-1178.

[8] Wang, F.; Yang, G.Q.; Xu, J.; Yu, W.W.; Shi, L.H.; Zeng, S.; Chen, L.Z.; Hu, D.Y.; Zhang, K.K. Simultaneous determination and method validation of clethodim and its

metabolites clethodim sulfoxide and clethodim sulfone in tobacco by LC-MS/MS, *Biomed. Chromatogr*. **2018**, 32, e4148.

[9] Du, P.Q., Wu, X.H., Xu, J., Dong, F.S., Liu, X.G., Zhang, Y., Zheng, Y.Q. Clomazone influence soil microbial community and soil nitrogen cycling, *Sci. Total. Environ.* **2018**, 644, 475-485.

[10] Zou, N., Han, Y.T., Li, Y.J., Qin, Y.H., Gu, K.J., Zhang, J.R., Pan, C.P., Li, X.S. Multiresidue Method for Determination of 183 Pesticide Residues in Leeks by Rapid Multiplug Filtration Purification and Gas Chromatography-Tandem Mass Spectrometry, *J. Agr. Food. Chem.*, **2015**, 64, 6061-6070.

[11] Zanella, R., Primel, E.G., Machado, S.L.O., Gonçalves, F.F., Marchezan, E. Monitoring of the herbicide clomazone in environmental water samples by solidphase extraction and high-performance liquid chromatography with ultraviolet detection. *Chromatographia*. **2002**, 55, 573–577.
